# Risks of radiation exposure to old military compasses with radioluminescent markings

**DOI:** 10.2478/aiht-2025-76-4038

**Published:** 2025-12-30

**Authors:** Igor Miklavčić, Igor Lassinger, Vanja Radolić, Marina Poje Sovilj

**Affiliations:** Josip Juraj Strossmayer University of Osijek, Department of Physics, Osijek, Croatia

**Keywords:** ambient dose equivalent rate, Bézard, gamma radiation, gamma spectrometry, M.49, M.53 S1, radioluminescence, radium, ambijentalni dozni ekvivalent, Bézard, gama zračenje, gama spektrometrija, M.49, M.53 S1, radioluminiscencija, radij

## Abstract

This study investigates the radiological safety of handling old radioluminescent military compasses whose markings are coated with radium-based luminous paint, historically used to ensure better visibility in dim or night-time conditions until the 1960s, when its use was discontinued and eventually banned due to the mounting evidence of its harmful health effects, including the increased risk of cancer. Using gamma spectrometry, we confirmed radium presence in three historical compass models, namely M.49, M.53 S1, and the small Bézard model. We also measured ambient dose equivalent rates to assess ionising radiation exposure at the usual distances from the source during routine handling or long-term storage of these devices. Our findings indicate that, under certain conditions, radiation doses may exceed the recommended safety limits for general public and underscore the importance of raising awareness about potential radiological risks associated with antique navigation instruments. Our study points to the need for appropriate safety protocols, handling procedures, and storage conditions to minimise these risks among collectors of military memorabilia as well as the general public in line with the ALARA principle.

## Abstract (hr)

Ovo istraživanje bavi se procjenom radiološkog rizika pri rukovanju starim vojnim kompasima s radioluminiscentnim oznakama, čije su oznake premazane luminiscentnom bojom na bazi radija. Takva se boja povijesno koristila radi bolje vidljivosti u uvjetima slabog osvjetljenja ili noću sve do 1960-ih godina, kada je njezina uporaba postupno ukinuta i naposljetku zabranjena zbog sve većeg broja dokaza o štetnim učincima na zdravlje, uključujući i povećan rizik od razvoja raka. Primjenom gama spektrometrije potvrđena je prisutnost radija u trima povijesnim modelima kompasa: M.49, M.53 S1 i u manjem modelu Bézard. Također su izmjerene vrijednosti ambijentalnog doznog ekvivalenta kako bi se procijenila izloženost ionizirajućem zračenju na uobičajenim udaljenostima od izvora tijekom redovitog rukovanja ili dugotrajnog pohranjivanja tih instrumenata. Rezultati pokazuju da u određenim uvjetima primljene doze zračenja mogu premašiti preporučene granične vrijednosti za opću populaciju od 1 mSv godišnje te naglašavaju važnost podizanja svijesti o mogućim radiološkim rizicima povezanima sa starim navigacijskim instrumentima. Ovo istraživanje upućuje na potrebu za primjenom odgovarajućih sigurnosnih protokola, postupaka rukovanja i uvjeta pohrane kako bi se ti rizici sveli na najmanju moguću mjeru među kolekcionarima vojnih memorabilija i u široj javnosti, u skladu s načelom ALARA (*As Low As Reasonably Achievable*).

In the early 20^th^ century, radium was used in paint mixtures applied to the markings of watches and compasses to make them to glow in the dark without requiring an external energy source (such as a battery). This phenomenon in which materials emit light as a result of energy absorbed from radioactive decay is known as radioluminescence. The colour (wavelength of the emitted photons) and intensity of this light can vary, depending on the combination of materials used and the conditions under which radioluminescence occurs. Common applications include dials, gunpoint sights, watches, and navigational instruments to improve visibility at night or in dim light conditions (e.g., murky water, fog). Radioluminescent materials were also used in emergency exit signs in buildings, industrial facilities, and aircrafts.

As radium’s harmful health effects came to light, its use was phased out and eventually banned in these applications, even though it remained in military use for quite some time.

In Croatia, as in other countries of the former Yugoslavia, it is still possible to find old military radioluminescent compasses, such as the handheld M.49, M.53 S1, M.53 S99 manufactured by Teleoptik (Zemun, Serbia), and two Bézard model compasses manufactured by G. Lufft Mess- und Regeltechnik GmbH (Stuttgart, Germany). These compasses are still in use by hikers, athletes, military and police academy trainees, and scouts. The instruction manuals for these compasses are mainly lost and do not warn about the presence of radium and the associated health risk.

Following media reports in neighbouring countries (1, 2), we investigated whether these compasses pose a health risk, either through potential radioactive contamination or exposure to ionising radiation in different usage and storage scenarios.

## MATERIALS AND METHODS

**Figure 1:**
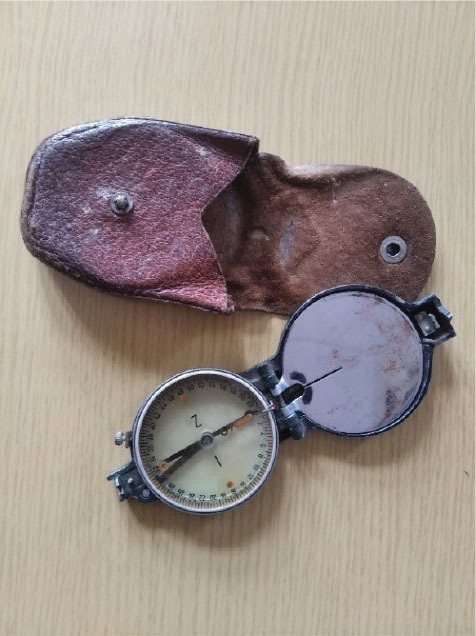
Handheld compass model M.49

**Figure 2:**
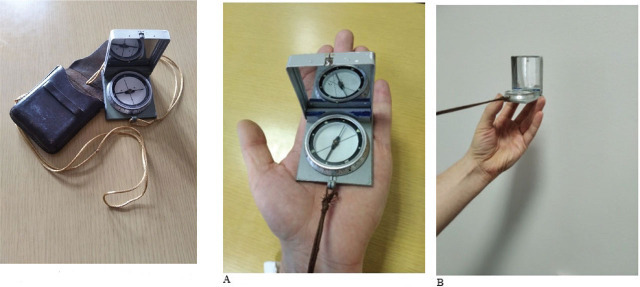
Handheld compass model M.53 S1

**Figure 3:**
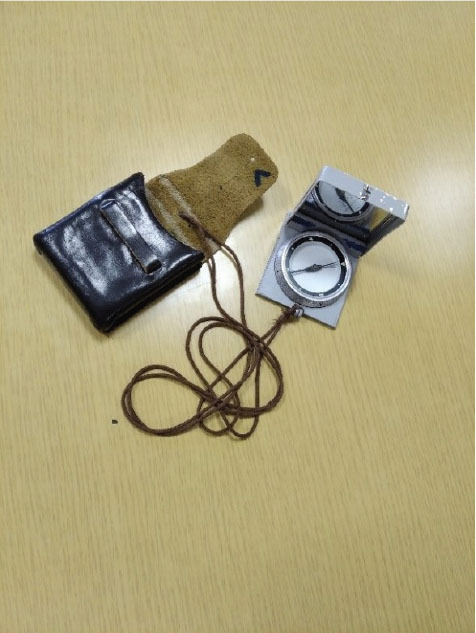
Handheld compass model M.53 S99

**Figure 4:**
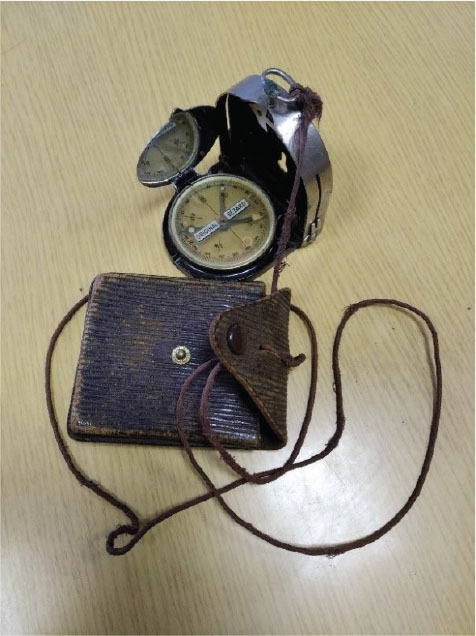
Handheld compass model Bézard (big)

**Figure 5:**
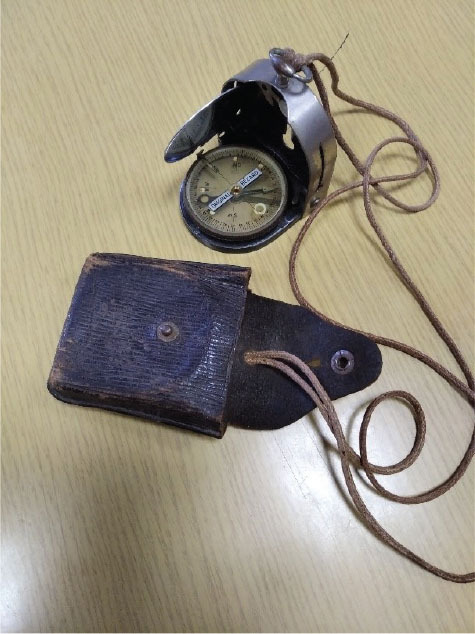
Handheld compass model Bézard (small)

To confirm the presence of radium in the compasses (M.49, M.53 S1, M.53 S99, and the two Bézard models named big and small hereinafter; Figures 1–5), we first used a portable, handheld Ipros 2 scintillation detector for gamma spectrometry equipped with a NaI(Tl) crystal (SCIONIX Holland BV, Bunnik, The Netherlands) (3).

Energy efficiency was calibrated on a ^137^Cs source with an activity of 37 kBq. It was relatively low, 7.5 % at the 662 keV photopeak over 360 s.

In order to assess potential contamination with radium originating from the paint, the compass surfaces were wiped with a cotton swab, and the swab analysed with the handheld gamma detector referred to above.

**Figure 6:**
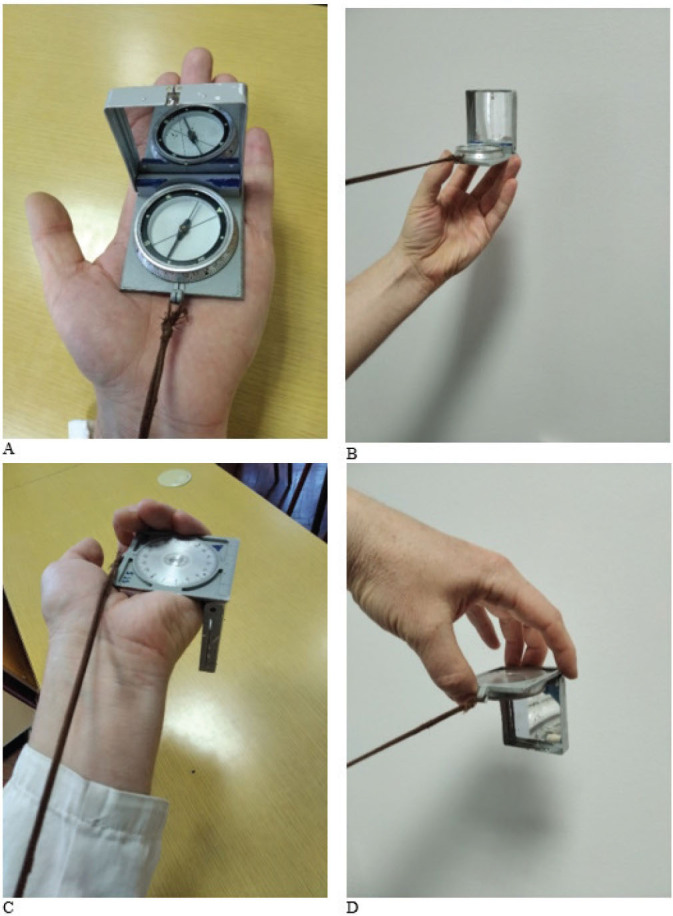
Specific hand positions for holding a compass during its use: a) determination of the main sides of the world; b) determination of the azimuth; c) reading the azimuth in degrees; and d) determination of the local angle below the horizon

Ambient dose equivalent rate H*(10) was measured over 15 min with a Thermo ESM FH40G-L10 digital radiation survey meter (Thermo Electron GmbH, Karlsruhe, Germany), which features a gamma detector with an integrated proportional counter and operates over an energy range from 30 keV to 4.4 MeV as described elsewhere (4). The instrument was configured to log data automatically at 10-second intervals in counter mode (measurement time per reading), resulting in 90 individual readings per position and distance. Mean and maximum values reported in the tables were calculated from these 90 measurements for each position. The positions of the compass and the detector were determined based on specific ways of holding and handling the compass as follows: keeping the compass closed inside the leather case, determining azimuth with the compass resting on the left palm, measuring local angles below the horizon with the compass inverted in the left hand, and determining angular distance with the compass closed and held between the fingers and thumb of the left hand (Figure 6).

Accordingly, ambient dose equivalent rates were labelled as follows: H_0_ – dose rate measured without the compass present (background); H_1(0 or 100)_ – dose rate for the compass in position 1 (bottom part of the compass inside the leather case); H_2(0 or 100)_ – dose rate for the compass in position 2 (top part of the compass inside the leather case); H_3(0 or 100)_ – dose rate for the compass in position 3 (bottom part of the compass outside the case); H_4(0 or 100)_ – dose rate for the compass in position 4 (top part of the compass without the cover). Designations in brackets, either 0 or 100, denote measurement distance from the source, namely 0 or 100 cm. These measurements are shown in Table 1.

**Table 1:** Measured ambient dose equivalent rates for the three compass models showing radium presence by measurement position and distance (means of 90 ten-second readings per position and distance)

**Measurement position (distance in cm)**	H*(10) (nSv/h)					
	**M.49**		**M.53 S1**		**Bézard (small)**	
	**Mean**	**Max**	**Mean**	**Max**	**Mean**	**Max**
H_0_ (background)	134	166	134	166	134	166
H_1(0)_	6070	7960	1260	1570	622	936
H_1(100)_	154	191	137	191	127	154
H_2(0)_	4630	5120	1230	1750	494	631
H_2(100)_	162	181	169	204	155	173
H_3(0)_	6620	8840	1470	1900	672	921
H_3(100)_	152	176	153	167	129	154
H_4(0)_	8140	9450	1830	2340	554	709
H_4(100)_	156	177	147	226	164	220

H_0_ – background dose rate without compass; H_1(0 or 100)_ – dose rate for the compass in position 1 (bottom part of the compass inside the leather case); H_2(0 or 100)_ – dose rate for the compass in position 2 (top part of the compass inside the leather case); H_3(0 or 100)_ – dose rate for the compass in position 3 (bottom part of the compass outside the case); H_4(0 or 100)_ – dose rate for the compass in position 4 (top part of the compass without the cover). Designations in brackets, either 0 or 100, denote measurement distance from the source, namely 0 or 100 cm

Ambient dose equivalent H*(10) has been recommended by the International Commission on Radiological Protection (ICRP) as an operational (measurable) quantity for assessing the effective dose E in the environment (5, 6). However, in the case of photon radiation from radium-painted compasses, H*(10) generally overestimates the effective dose E, as it is a dose equivalent at a depth of 10 mm within the 30 cm sphere defined by the International Commission on Radiation Units and Measurements (so called ICRU sphere) rather than in an anthropomorphic phantom. According to ICRP 116 (7), for photon energies in the range of several-hundred keVs emitted by ^226^Ra progenies, the E/H*(10) ratio is typically about 0.6 for broad-beam or whole-body irradiation geometries. Based on the measured H*(10), we therefore estimated the effective dose rate using the following equation:

[1] $$\text{E}/\text{t}={\text{H}}^{*}\left(10\right)/\text{t}\times 0.6$$

To estimate the annual effective dose E, we multiplied the effective dose rate with the time of exposure as follows:

[2] $$\text{E}=\text{E}/\text{t}\times {\text{t}}_{\text{exposure}}$$

The annual effective dose estimates included two scenarios: two hours of daily use over the entire year and a more realistic estimation assuming a 40-hour use per year (e.g., during orienteering exercises, competitions, or skill training).

Before analysing the samples, a gamma spectrum of the laboratory background radiation was recorded in order to identify any additional radionuclides present in the environment and to ensure accurate interpretation of the compass spectra. In addition, the indoor radon concentration in the laboratory was measured using solid-state nuclear track-etched detectors over a one-year exposure period to verify that radon and its progeny did not contribute significantly to the recorded gamma background.

## RESULTS AND DISCUSSION

**Figure 7:**
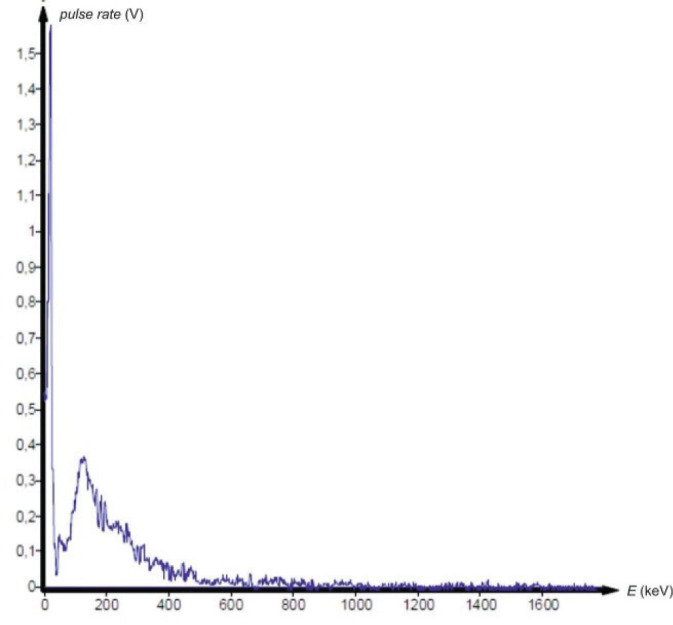
Background spectrum

**Figure 8:**
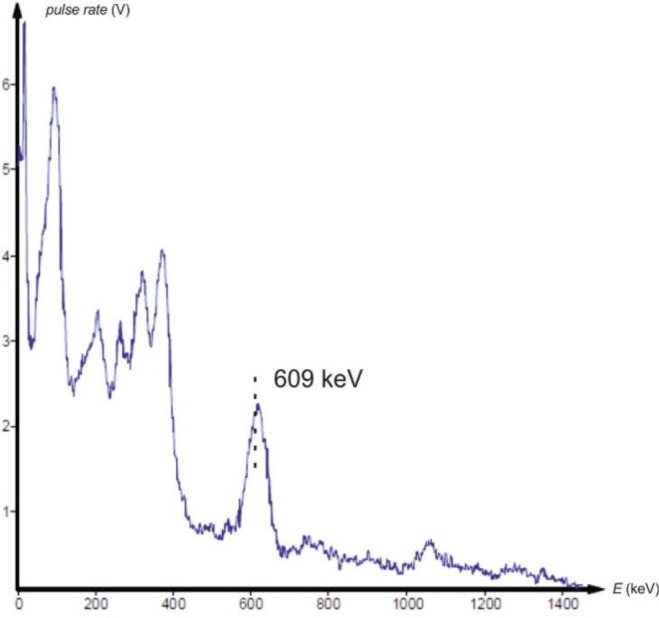
Gamma spectrum of the handheld compass model M.49

**Figure 9:**
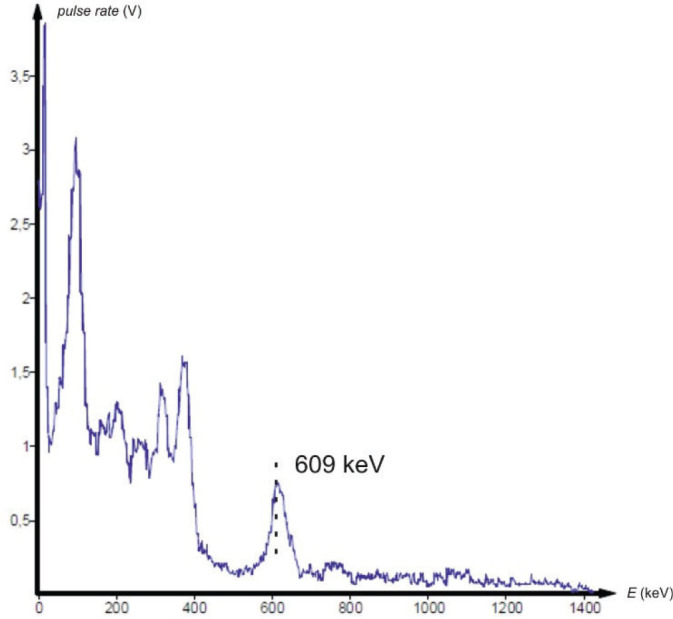
Gamma spectrum of the handheld compass model M.53 S1

**Figure 10:**
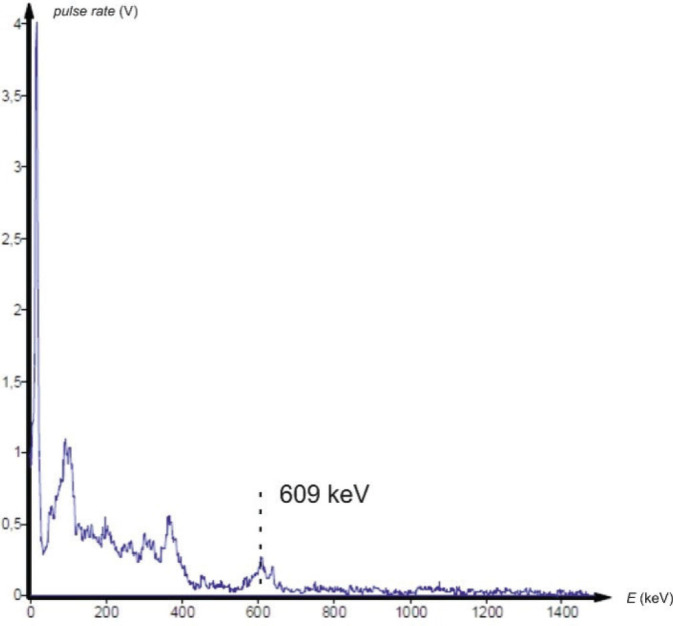
Gamma spectrum of the handheld compass model Bézard (small)

Gamma spectrometry showed no background presence of radionuclides in the lab environment (Figure 7), and the measured indoor radon activity concentration was 30±11 Bq/m^3^, which points to a negligible contribution from airborne radon and its progeny (8). However, it confirmed the presence of radium-based radioluminescent paint in three compass models: M.49, M.53 S1, and Bézard (small). In their spectra, we observed distinct gamma peaks associated with the ^226^Ra decay chain (Figures 8–10). The 186 keV line is the only one directly attributable to ^226^Ra decay and therefore the primary indicator of radium presence. The other gamma lines observed in the spectra arise from the radioactive decay of ^226^Ra to radon gas (^222^Rn), whose further decay produces short-lived metallic radionuclides ^214^Pb and ^214^Bi, the first emitting 295 keV and 352 keV and the second 609 keV gamma rays, clearly visible on the recorded spectra. A combined feature around 80 keV, arising from Kα fluorescent X-rays and low-energy gamma emissions from ^214^Bi (77.1 keV) and ^214^Po (79.3 keV), further confirms the presence of the radium decay chain progenies. These daughter-product peaks appear only when ^226^Ra is present, as they require continuous in-paint production of ^222^Rn (9, 10).

**Figure 11:**
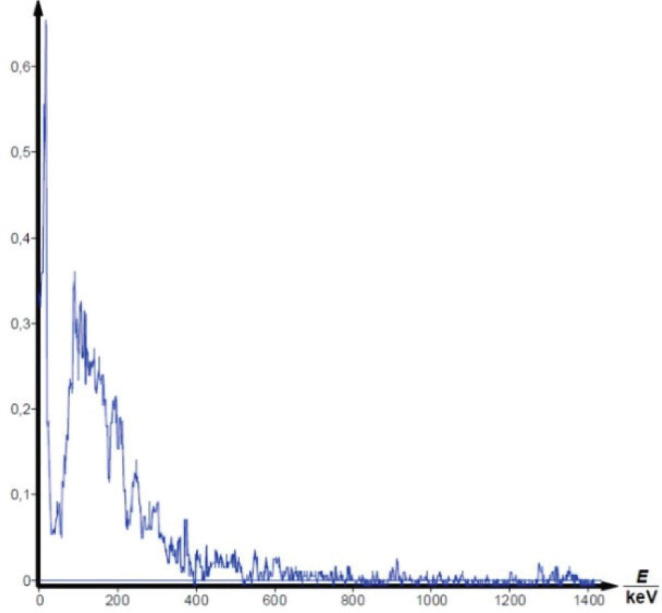
Gamma spectrum of the handheld compass model M.53 S99

**Figure 12:**
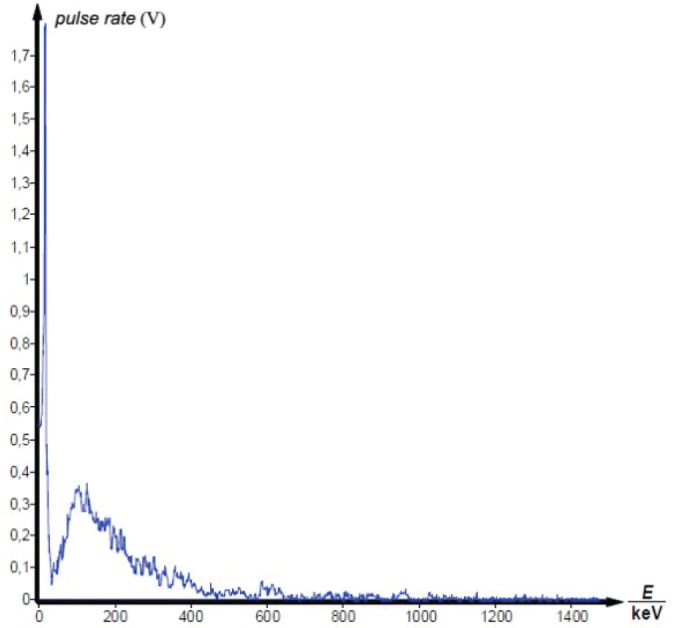
Gamma spectrum of the handheld compass model Bézard (big)

In contrast, the M.53 S99 and Bézard (big) models exhibited only characteristic fluorescence peaks (notably the 74.2 keV Kα line) without identifiable gamma emissions from the ^226^Ra series (Figures 11 and 12).

**Figure 13:**
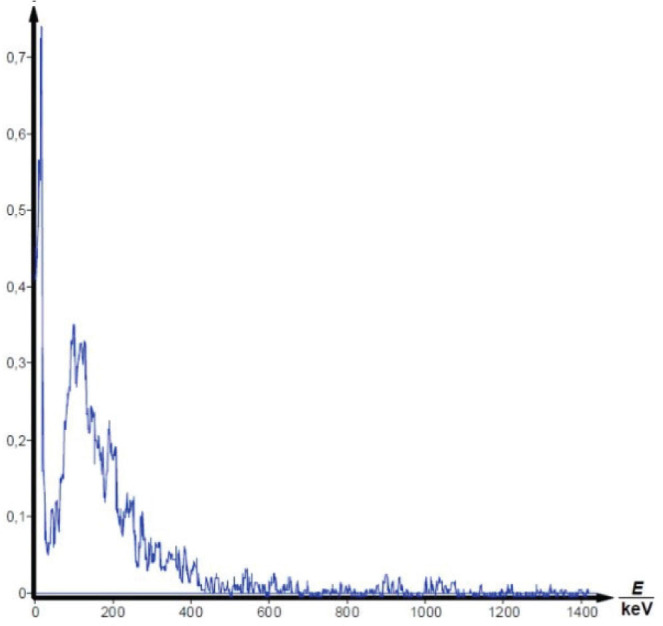
Gamma spectrum of the cotton swab used to wipe the surfaces of the handheld compasses with confirmed presence of radioluminescent paint

Swab sample analysis with the gamma detector yielded no detectable radionuclides, suggesting the absence of surface contamination (Figure 13).

Table 1 summarises the mean and maximum ambient dose equivalent rates H*(10) obtained for the three radium-positive compass models across different handling positions and at distances of 0 cm (contact) and 100 cm. Each value represents the mean or maximum of 90 readings recorded over 15 min per position.

Table 2 shows that the calculated annual effective dose emitted by the three compass models ranges from 0.02 to 2.6 mSv, depending on either of the two scenarios. For the 2 h/day scenario, the estimated annual effective doses range from 0.39 mSv to 2.6 mSv, depending on the compass model. For the 40 h/year scenario, the range is 0.02–0.14 mSv. For the shelved compasses, the annual estimated dose at 1 m distance is 0.63 mSv. According to the ICRP (5), the annual dose limit for members of the general public is 1 mSv (excluding natural background and medical exposure). The M.49 compass clearly exceeds the 1 mSv annual limit in the 2 h/day scenario, while the M.53 S1 and Bézard (small) remain below or close to this limit. Occasional handling (the 40 h/year scenario) results in doses far below the annual limit and therefore does not pose a significant radiological risk.

**Table 2:** Calculated annual effective dose while using the compass in the left-hand position for two scenarios: frequent (daily) use and occasional use (in a very conservative estimation)

**Annual effective dose E (mSv)**	**M.49**		**M.53 S1**		**Bézard (small model)**	
	**2 h/day**	**40 h/year**	**2 h/day**	**40 h/year**	**2 h/day**	**40 h/year**
	2.6*	0.14	0.66	0.04	0.39	0.02

* – exceeding the ICRP limit of 1 mSv/year for the general public (5)

Our results are consistent with those of Gillmore et al. (11), who reported dose rates of up to 10 µSv/h from vintage radium-dial watches in the United Kingdom. These findings reinforce the view that such artefacts can still contribute to doses that approach or exceed the 1 mSv/year exposure limit if handled daily or stored in bulk.

## CONCLUSION

Our study has several limitations, including a limited number of compass models examined and the absence of radon emanation measurements, which would provide a more comprehensive assessment of potential inhalation exposure pathways. Since ^226^Ra is an alpha emitter and the parent radionuclide of radon (^222^Rn), future analyses should include radon emanation and progeny accumulation using a radon detector or alpha-spectrometric system in a container of known volume. This approach would enable the estimation of radon release rates from luminous paint and evaluation of potential inhalation doses in poorly ventilated storage environments.

Despite its limitations, our findings clearly confirm the presence of radium-based radioluminescent paint in vintage military models M.49, M.53 S1, and Bézard small and point to effective doses of concern. While short-term, occasional use is unlikely to cause acute health effects, scenarios involving prolonged handling, storage in confined spaces, or the accumulation of multiple radium-containing items may significantly increase external dose as well as the risks of inhaling radon and its progeny. The potential for elevated exposure in storage environments containing larger numbers of such devices will be addressed in future investigations.

As radium’s long half-life means these risks shall persist for centuries, it is essential for the owners, collectors, safekeepers, and users of these items to adopt appropriate safety protocols such as wearing gloves, using ventilated storage, labelling, limiting access, and performing periodic radiation monitoring. These measures should be aligned with international radiation protection principles to reduce exposure to “As Low As Reasonably Achievable” (ALARA), safeguarding both collectors and the public while preserving historical artefacts.
